# Levodopa–Entacapone–Carbidopa Intrajejunal Infusion in Advanced Parkinson's Disease – Interim Analysis of the ELEGANCE Study

**DOI:** 10.1002/mdc3.70046

**Published:** 2025-03-25

**Authors:** Daniel Weiss, Wolfgang H. Jost, József Attila Szász, Zvezdan Pirtošek, Ivan Milanov, Volker Tomantschger, Norbert Kovács, Harry Staines, Bharat Amlani, Niall Smith, Teus van Laar

**Affiliations:** ^1^ Department for Neurodegenerative Diseases Centre for Neurology, Hertie‐Institute for Clinical Brain Research Tübingen Germany; ^2^ Parkinson‐Klinik Ortenau Wolfach Germany; ^3^ Department of Neurology, “George Emil Palade” University of Medicine Pharmacy, Science and Technology Târgu Mureș Romania; ^4^ Department of Neurology University Medical Centre and Faculty of Medicine Ljubljana Slovenia; ^5^ University Neurological Hospital “St. Naum” Sofia Bulgaria; ^6^ Gailtal‐Klinik Hermagor Neurorehabilitation Austria; ^7^ Department of Neurology, Medical School University of Pécs Baranya Hungary; ^8^ Sigma Statistical Services Balmullo UK; ^9^ Britannia Pharmaceuticals Limited Reading UK; ^10^ Parkinson Expertise Center at University of Groningen Groningen The Netherlands

**Keywords:** advanced Parkinson's disease, levodopa, entacapone, carbidopa intestinal gel, clinical practice

## Abstract

**Background:**

Levodopa–entacapone–carbidopa intestinal gel (LECIG) was introduced in 2018 as a device‐aided therapy for advanced Parkinson's disease (PD).

**Objectives:**

The ELEGANCE study (NCT05043103) is gathering real‐world data on long‐term efficacy, safety and patient‐reported outcomes with LECIG from 13 European countries. This article reports data from the planned interim analysis.

**Methods:**

The study enrolled patients prescribed LECIG as part of routine clinical care. We evaluated patients at V1 before starting LECIG treatment (in seven patients V1 data were obtained retrospectively), and thereafter at V2 (3–6 months) or V3 (6–12 months).

**Results:**

This analysis includes 167 patients from 37 centers. Three patients from this analysis set (1.8%) discontinued the study. Mean (±SD) daily OFF‐time hours (MDS‐UPDRS IV item 4.3) were substantially reduced by 3.47 ± 3.56 h at V2 (baseline: 5.15 ± 3.05; *P* < 0.0001). Similarly, MDS‐UPDRS part IV total scores were reduced by 4.24 ± 4.08 at V2 (baseline: 10.77 ± 3.83); (*P* = 0.0001) and MDS‐UPDRS part II scores by 3.63 ± 7.76 at V2 (baseline: 20.65 ± 8.17; *P* = 0.0004). PDSS‐2 total scores were sustainably improved (reduction of 7.38 ± 10.72 at V2 [baseline: 25.21 ± 10.62]; *P* < 0.0001), as was the PDQ‐8 summary index score indicating an improvement in quality of life (QoL) (reduction of 13.3 ± 19.05 at V2 [baseline: 46.34 ± 20.09]; *P* < 0.0001). For all parameters improvements were maintained at V3. Patient‐reported satisfaction with the LECIG pump was high. Most adverse events were related to the procedure or the device.

**Conclusions:**

Routine use of LECIG for up to 12 months provided sustained control of motor symptoms, and was well tolerated with a positive impact on QoL and high patient satisfaction.

Oral dopamine replacement therapy is an effective treatment for people with Parkinson's disease (PD). However, due to progressive dopaminergic denervation and disease progression, motor and non‐motor fluctuations, including wearing off and dyskinesias, develop in the majority of patients, despite efforts to optimize therapy with adjunctive oral medications.^1^, ^2^, ^3^, ^4^ Gastroparesis, which commonly occurs in patients with PD, may further complicate the transport and resorption of oral levodopa, and intensive oral medication regimens represent a considerable burden to patients and their caregivers.^5^

Device‐aided dopamine replacement therapy provides continuous, rather than pulsatile, dopaminergic stimulation. Until recently, this comprised continuous subcutaneous apomorphine (APO) infusion and levodopa–carbidopa intestinal gel (LCIG). Both have been demonstrated in randomized controlled and open‐label trials to stabilize dopaminergic motor fluctuations by reducing both the time of uncontrolled OFF symptoms and ON state with troublesome dyskinesias.^6^, ^7^, ^8^, ^9^ This contributed to improvement in quality of life and activities of daily living.^10^ Nevertheless, therapeutic alternatives are required to meet the wide range of patient needs including tolerability or device‐related issues. In real‐world clinical practice up to 43% of patients still receive adjunctive oral therapy despite being treated with LCIG infusion,^11^ and some centers prefer to add an oral catechol‐O‐methyltransferase (COMT) inhibitor to spare levodopa dosing and potentially to save costs.^12^, ^13^, ^14^

Levodopa–entacapone–carbidopa intestinal gel (LECIG) infusion (LECIGON®, Britannia Pharmaceuticals Ltd.) was introduced in Sweden in 2018,^15^, ^16^, ^17^ and since then has received marketing authorization in several European countries. The addition of entacapone to the LECIG formulation increases the bioavailability of levodopa compared with LCIG, allowing a lower levodopa dose, and hence lower infusion volume, to be administered.^18^ This has made it possible to reduce the size and the weight of the infusion pump compared to that for LCIG infusion. In addition, the lower levodopa dose means that degradation to 3‐O‐methyldopa (3‐OMD)—catalyzed by COMT and depending on vitamin B12—is reduced by approximately 35%^18^ independent of the COMT rs4680 polymorphism.^14^ Whether COMT‐inhibitors counteract the development of peripheral neuropathy has not been confirmed in controlled studies^15^, ^16^, ^19^ but has been raised as a hypothesis in a retrospective study.^20^

Real‐world clinical experience with LECIG is still limited, with data predominantly derived from smaller, single‐center studies in Sweden (24 patients), Romania (20 patients) and Bulgaria (five patients).^21^, ^22^, ^23^ Retrospective analyses from 21 centers in Spain (73 patients), 12 centers in Romania (74 patients) and a single center in Finland (30 patients)^24^, ^25^, ^26^ have reported improvement in motor function. Significant reductions have been reported in daily OFF time, duration and severity of dyskinesias, as well as in the use of concomitant oral PD medications. In addition, the reports suggested that LECIG has a similar safety and tolerability profile to that seen with LCIG. The Swedish study noted that patients switching from LCIG preferred the smaller size and lighter weight of the LECIG pump.

To add to this evidence base, the ELEGANCE study (Global Long‐Term Registry on Efficacy and Safety of Lecigon in Patients with Advanced Parkinson's Disease in Routine Care) has been established across 13 European countries. The study aims to document real‐world information from a large cohort of advanced PD patients when experiencing motor fluctuations that remain uncontrolled despite optimized oral/transdermal dopamine replacement therapy.^15^ The efficacy and safety data will be enriched with information from patient‐reported outcomes. Here, we report interim findings on patients who have been treated with LECIG for up to 12 months.

## Methods

ELEGANCE (NCT05043103) is an ongoing, 24‐month, non‐interventional, observational study to evaluate the long‐term effectiveness and safety of LECIG in patients with advanced PD in routine care. The study commenced recruitment in July 2021 and when recruitment was completed in September 2024, 312 patients had been enrolled into the registry. This planned interim analysis presents the initial observational efficacy, safety and patient‐reported outcomes for patients who have available data for up to 12 months of treatment.

### Inclusion and Exclusion Criteria

ELEGANCE study centers offered study participation to adult patients (≥18 years of age) with advanced PD with motor fluctuations, including uncontrolled OFF‐periods or dyskinesia, despite taking optimized oral/transdermal PD therapy and who had been prescribed LECIG treatment as part of routine clinical practice, in accordance with the Summary of Product Characteristics (SmPC).^27^ Patients switching from both oral dopamine replacement therapy and another infusion therapy were considered. Patients were excluded if they had contraindications to LECIG treatment, as defined in the current SmPC^27^. Patients should not have been taking part in an interventional clinical trial at the same time. Furthermore, acute severe illness of any cause rendering them unable to manage the infusion device, or poor compliance due to severe dementia, agitation or alcohol abuse, led to exclusion. All patients provided written informed consent before enrolment in accordance with the Declaration of Helsinki. Enrolment was possible either before or after LECIG was started, but no longer than 3 months after treatment initiation.

### Study Visits and Patient Follow‐Up

Observations of patients enrolled in the study were undertaken at an initial assessment visit (V1; referred to here as “baseline” for simplicity) prior to starting LECIG treatment. Patients could be enrolled into the trial by providing consent up to 3 months after their initial titration onto LECIG. This applied to seven patients, in whom V1 measurements were obtained from the clinical records. These data referred to the time period prior to starting LECIG and were drawn retrospectively from the records during the initial titration of LECIG no longer than 7 days after commencing therapy. Follow‐up visits in accordance with clinical practice took place after approximately 3–6 months of treatment (V2), 6–12 months of treatment (V3), and then every 6 months thereafter up to 24 months of LECIG treatment. In this interim analysis, we report observations up to V3.

### Outcome Measures

The full range of outcome measures can be found at www.clinicaltrials.gov/study/NCT05043103. This interim analysis reports data for the following variables: patient demographics (age, sex) and disease characteristics (duration of PD, age at diagnosis, whether they switched to LECIG from oral PD therapy or another infusion therapy). Daily levodopa dose (mg/day) administered through LECIG treatment was calculated as follows: morning dose (mg) plus maintenance dose (mg/hour) × infusion hours. Results are presented by visit. Further variables were recorded at V1 to V3 if they were represented in the standard of care for patients with advanced PD in the participating centers. Daily hours of OFF time and percentage of the waking day spent in the OFF state were assessed based on the Movement Disorder Society Unified Parkinson's Disease Rating Scale (MDS‐UPDRS) part IV scores for item 4.3.^28^ Daily hours spent with dyskinesias were recorded using MDS‐UPDRS part IV question 4.1. Scores for MDS‐UPDRS part II were also assessed.^28^ Nocturnal sleep problems were evaluated using the Parkinson's Disease Sleep Scale 2 (PDSS‐2).^29^

Quality of life was assessed using either the 8‐item Parkinson's Disease Questionnaire (PDQ‐8) or extracted from the 39‐item (PDQ‐39).^30^, ^31^ The clinician's assessment (CGI‐I),^32^ and the patient's assessment of treatment (PGI‐I)^33^ were measured using the Clinical Global Impression of Improvement scale (a seven‐point scale: Compared to the patient's condition at admission to the project [prior to medication initiation], this patient's condition is: 1 = very much improved to 7 = very much worse), respectively.

From V2 onwards, patient‐reported satisfaction with the infusion device (size, weight, noise, handling of and overall satisfaction with the pump, each scored on a visual analogue scale of 1 = absolutely dissatisfied to 10 = absolutely satisfied) was recorded for these five parameters as well as their total sum score. Whether patients were using the multi‐rate programming function of the infusion pump was also noted.

### Safety

Treatment‐emergent adverse events (AEs), including serious AEs (SAEs), that occurred at any time during the course of LECIG treatment were reported. Study sites were encouraged to report all observed AEs. In each case these were categorized according to whether they were related to the procedure, the device, or to the drug treatment. In addition, data on AEs of particular relevance to LECIG were analyzed based on the Medical Dictionary for Regulatory Activities (MedDRA) “preferred terms”. These included dopaminergic side effects, occurrence of peripheral neuropathy, vitamin B12 deficiency, and diarrhea.

### Definition of Data Sets

As of December 4, 2023, the cut‐off date for the interim analysis, the study had enrolled 245 patients. Of these, 168 patients with a visit recorded at V2 or V3 were considered in this analysis. Safety endpoints were analyzed using the Safety Set comprising the 167 patients treated with LECIG. The full analysis set (FAS) refers to LECIG‐treated patients with at least one measure of effectiveness at V2 (3–6 months of treatment) or V3 (6–12 months of treatment) (*n* = 167). All patients in the Safety Set were included in the FAS. The final analysis of ELEGANCE will report on the full cohort of patients, including those who did not report any effectiveness measures after baseline.

### Statistical Analysis

Here we report the exploratory interim findings of an uncontrolled, observational study. No sample size calculation was performed; therefore, the findings and p‐values are not intended to be interpreted with confirmatory intent. Categorical data are presented by frequency and the percentage of available data. Data analyzed as continuous variables are presented using the mean with standard deviation or 95% confidence interval assuming normality. The one‐sample t‐test is used to test for a significant change at post‐baseline visits (V2, V3) from baseline (V1). No missing data have been imputed; however, partially missing dates have been imputed by the start and end of the known month. Owing to the exploratory nature of this study, no adjustment for multiplicity has been performed. Statistical analyses were done with SAS version 9.4. Tests with a two‐sided significance level of less than 5% are defined as significant.

## Results

### Patient Population

163 of 167 patients in the FAS were Caucasian. The median follow‐up time was 182 days (quartile 1:136 days; quartile 3:314 days). Full demographics and disease characteristics of the FAS are shown in Table 1. The majority of patients were in modified Hoehn and Yahr stages 3 (54 patients, 32.9%) or 4 (75 patients, 45.7%) at the initial assessment visit (V1) and exhibited significant motor fluctuations with a mean of 5.15 hours of uncontrolled daily OFF time. The majority of patients switched from oral dopamine replacement therapy to LECIG, while 22 patients switched from either LCIG (*n* = 14) or APO (*n* = 8) infusion therapy. Reasons for switching from other device‐aided infusion therapies were perceived lack of efficacy (*n* = 12), adverse events (*n* = 4) or poor compliance (*n* = 1) with current treatment, a desire to use the smaller LECIG pump (*n* = 4), and an unrelated surgical procedure (*n* = 1).

**TABLE 1:** Pre‐LECIG treatment demographics and disease characteristics for the 167 patients with advanced Parkinson's disease included in the analysis

Parameter	Baseline value
Age, years (mean ± SD)	68.2 ± 7.7
Sex	59% male, 41% female
Duration of PD, years (mean ± SD)	13.3 ± 6.3
Age at diagnosis, years (mean ± SD)	55 ± 9.6
Switched from oral therapy, *n* (%)	145 (86.8)
Switched from another device‐aided infusion therapy, *n* (%)	22 (13.2)
Daily hours of OFF time (mean ± SD score for MDS‐UPDRS part IV, question 4.3) (*n* = 128)	5.15 ± 3.05
Percentage of waking day spent in the OFF state (mean ± SD score for MDS‐UPDRS part IV, question 4.3) (*n* = 142)	1.82 ± 0.84, equivalent to 25%–50%
MDS‐UPDRS part II (Motor Aspects of Experiences of Daily Living) total score (mean ± SD) (*n* = 78)	20.65 ± 8.17
Patients with dementia, *n* (%)	37 (22.6) (21 mild; 15 moderate; 1 severe)
Patients with a history of hallucinations or psychosis, *n* (%)	16 (10.1)

Abbreviations: LECIG, levodopa–carbidopa–entacapone intestinal gel; MDS‐UPDRS, Movement Disorder Society Unified Parkinson's Disease Rating Scale; PD, Parkinson's disease; SD, standard deviation.

In this dataset, three patients (1.8%) discontinued the study before or at V3. One patient had an AE of “delusion” which led to withdrawal of LECIG treatment and study discontinuation. The other two patients did not report any AE during the study and were receiving LECIG at the time of early discontinuation. One of these patients withdrew consent for unknown reasons, the other due to changing to an unknown PD therapy.

### LECIG Treatment

Mean (±SD) LECIG treatment duration for the 167 patients (time between the first day of LECIG treatment to the day of the last visit used in the analyses) was 217.7 ± 100.5 days with a median of 182 days (range: 76 to 592 days). This equates to a cumulative LECIG exposure of 99.5 years. The daily continuous infusion duration at the start of LECIG treatment was 15.95 ± 2.55 hours and remained stable over time. The total daily levodopa dose of LECIG infusion was 886.8 ± 300.3 mg/day (*n* = 163) at the start of LECIG treatment, 914.9 ± 294.1 mg/day at V2 (*n* = 152), and 968.3 ± 312.3 mg/day at V3 (*n* = 72). A total of 54 patients (32.3%) used the multi‐rate programming function. Of these, 47 patients (28.1%) used two different flow rates at least once up to V3 and 11 patients (6.6%) used three different flow rates at least once. Across visits, few patients used 24‐h continuous infusion (4.9%, 5.9%, 6.9%; V1 to V3, respectively), and some patients used continuous infusion therapy for more than 16 h daily (17.1%, 17.8%, 15.3%, respectively).

### Efficacy Outcomes

**Figure 1:**
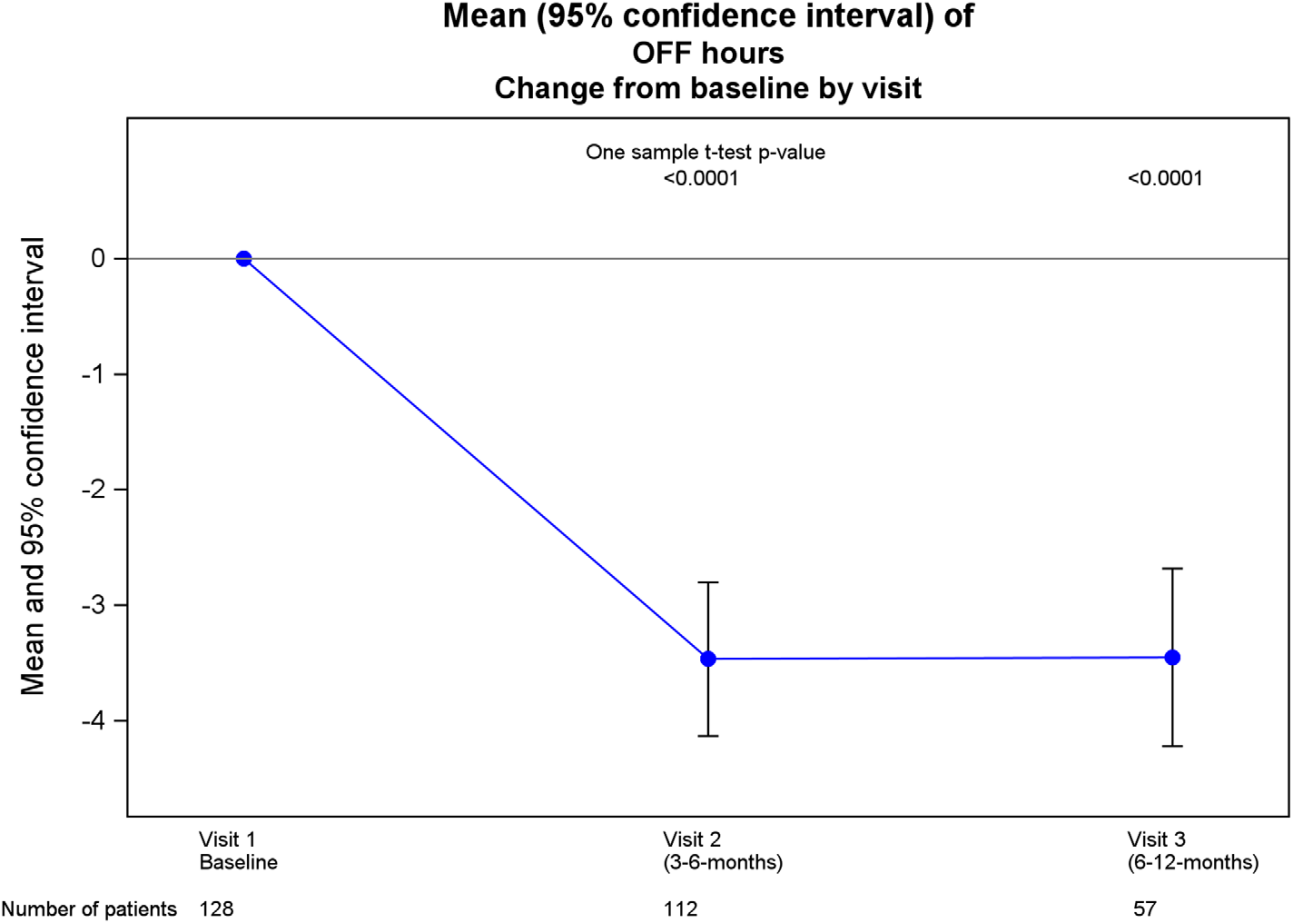
Daily hours of OFF time by visit. Data are mean ± 95% confidence interval.

**Figure 2:**
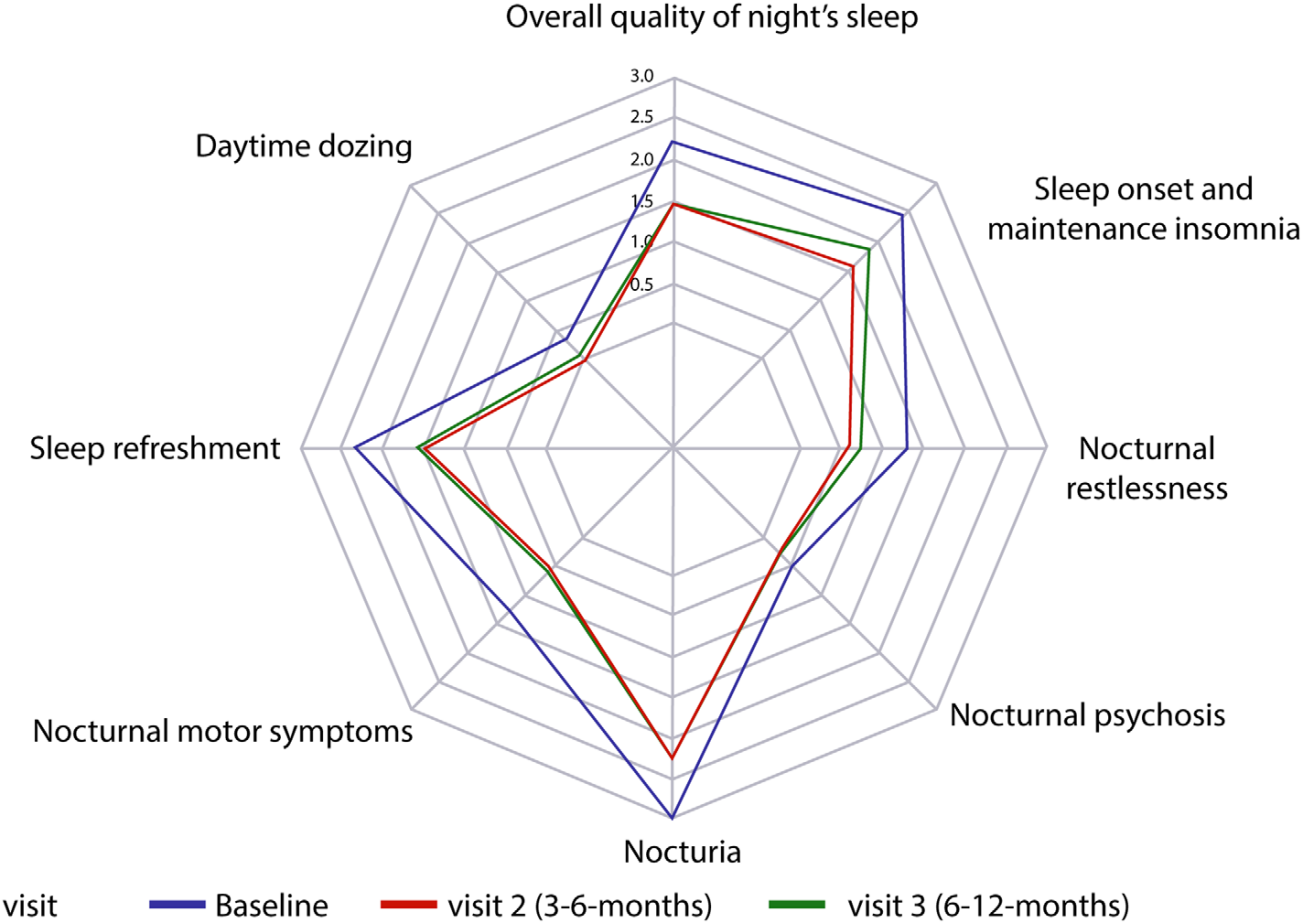
Radar plot indicating the mean values per visit of the clinically meaningful sleep subdomains from the PDSS‐2 score. A scale from 0 to 4 is used with lower numbers associated with better outcome. Items were grouped according to the APOMORPHEE study^36^: overall quality of night's sleep (item 1), sleep onset and maintenance insomnia (items 2 and 3), nocturnal restlessness (items 4 and 5), nocturnal psychosis (items 6 and 7), nocturia (items 8 and 9), nocturnal motor symptoms (items 10–13), sleep refreshment (item 14), and daytime dozing (item 15).

Efficacy outcomes are summarized in Table 2. Daily hours of OFF time were substantially reduced by V2 (after 3–6 months of treatment) compared with baseline values (*P* < 0.0001) and this reduction was sustained at V3 (after 6–12 months of treatment) (Fig. 1). Similar reductions in daily OFF time from baseline were seen regardless of whether patients switched from oral medication or from an infusion therapy (Table 2; Table S1). Both MDS‐UPDRS part II and MDS‐UPDRS part IV total scores were improved at V2 and V3 compared with baseline suggesting clinically meaningful overall improvements in functional ability and motor fluctuations exceeding the minimal clinically important difference thresholds.^34^, ^35^ Time spent in a dyskinetic ON state decreased at V2 and V3 compared to baseline (Table 2). PDSS‐2 total sleep scores, as well as the individual domains of “motor symptoms at night”, “PD symptoms at night”, and “disturbed sleep” improved from the initial assessment visit at both V2 and V3 (Table 2), suggesting an improvement in overall sleep quality. In addition, we have provided a more detailed representation of the different clinically meaningful sleep subdomains from the PDSS‐2 score and grouped them as suggested from the APOMORPHEE study^36^ (Fig. 2).

**TABLE 2:** Change from baseline in efficacy parameters following LECIG treatment for up to 12 months

Parameter	Pre‐LECIG treatment value (baseline)	Change from pre‐LECIG treatment at visit 2 (3–6 months)	*P*‐value	Change from pre‐LECIG treatment at visit 3 (6–12 months)	*P*‐value
Daily hours of OFF time: mean ± SD score for MDS‐UPDRS part IV (Motor Complications), question 4.3					
All patients	5.15 ± 3.05 (*n* = 128)	−3.47 ± 3.56 (*n* = 112)	*P* < 0.0001	−3.45 ± 2.90 (*n* = 57)	*P* < 0.0001
Switched from oral therapy	5.22 ± 2.97 (*n* = 112)	−3.53 ± 3.31 (*n* = 99)	–	–	–
Switched from an infusion therapy	4.69 ± 3.60 (*n* = 16)	−3.00 ± 5.20 (*n* = 13)	–	–	–
Percentage of the waking day spent in the OFF state*: mean ± SD score for MDS‐UPDRS part IV (Motor Complications), question 4.3					
All patients	1.82 ± 0.84 (*n* = 142) 25–50%*	−0.87 ± 1.01 (*n* = 127) 0–25%*	*P* < 0.0001	−0.92 ± 0.76 (*n* = 61) 0–25%*	*P* < 0.0001
Switched from oral therapy	1.82 ± 0.83 (*n* = 125) 25–50%*	−0.87 ± 0.96 (*n* = 113) 0–25%*	–	–	–
Switched from an infusion therapy	1.82 ± 0.95 (*n* = 17) 25–50%*	−0.86 ± 1.41 (*n* = 14) 0–25%*	–	–	–
Daily hours spent with dyskinesias: mean ± SD score for MDS‐UPDRS part IV (Motor Complications) question 4.1					
All patients	3.67 ± 3.46 (*n* = 126)	−1.55 ± 3.99 (*n* = 111)	*P* < 0.0001	−1.19 ± 4.14 (*n* = 53)	0.0417
Switched from oral therapy	5.22 ± 3.83 (*n* = 9)	−0.64 ± 6.50 (*n* = 7)		−3.25 ± 8.13 (*n* = 2)	
Switched from an infusion therapy	3.42 ± 4.01 (*n* = 6)	1.42 ± 8.42 (*n* = 6)		1.00 (*n* = 1)	
MDS‐UPDRS part IV (Motor Complications) total score (mean ± SD)					
All patients	10.77 ± 3.83 (*n* = 138)	−4.24 ± 4.08 (*n* = 123)	*P* < 0.0001	−3.77 ± 3.83 (*n* = 60)	*P* < 0.0001
Switched from oral therapy	10.62 ± 3.85 (*n* = 121)	−4.28 ± 4.11 (*n* = 109)	–	–	–
Switched from an infusion therapy	11.82 ± 3.63 (*n* = 17)	−4.00 ± 3.92 (*n* = 14)	–	–	–
MDS‐UPDRS part II (Motor Aspects of Experiences of Daily Living) total score (mean ± SD)					
All patients	20.65 ± 8.17 (*n* = 78)	−3.63 ± 7.76 (*n* = 65)	*P* = 0.0004	−3.38 ± 7.37 (*n* = 29)	*P* = 0.0198
Switched from oral therapy	20.39 ± 7.64 (*n* = 67)	−3.65 ± 7.21 (*n* = 54)	–	–	–
Switched from an infusion therapy	22.27 ± 11.20 (*n* = 11)	−3.55 ± 10.47 (*n* = 11)	–	–	–
PDSS‐2 scores (mean ± SD)					
Total score	25.21 ± 10.62 (*n* = 110)	−7.38 ± 10.72 (*n* = 100)	*P* < 0.0001	−6.63 ± 9.45 (*n* = 41)	*P* < 0.0001
Motor symptoms at night	6.71 ± 4.51 (*n* = 110)	−2.23 ± 4.70 (*n* = 102)	*P* < 0.0001	−2.80 ± 4.03 (*n* = 41)	*P* < 0.0001
PD symptoms at night	6.78 ± 3.93 (*n* = 110)	−2.07 ± 3.83 (*n* = 100)	*P* < 0.0001	−2.15 ± 3.53 (*n* = 41)	*P* = 0.0004
Disturbed sleep	11.72 ± 4.42 (*n* = 110)	−2.92 ± 4.85 (*n* = 102)	*P* < 0.0001	−1.68 ± 4.58 (*n* = 41)	*P* = 0.0238
PDQ‐8 summary index scores (mean ± SD)					
Total score	46.34 ± 20.09 (*n* = 135)	−13.3 ± 19.05 (*n* = 127)	*P* < 0.0001	−11.1 ± 16.33 (*n* = 52)	*P* < 0.0001

Abbreviations: LECIG, levodopa–carbidopa–entacapone intestinal gel; MDS‐UPDRS, Movement Disorder Society Unified Parkinson's Disease Rating Scale; PD, Parkinson's disease; PDSS‐2, Parkinson's Disease Sleep Scale 2; SD, standard deviation. The Minimal Clinically Important threshold values for improvement judging clinical relevance are −3.05 for MDS‐UPDRS Part II,^34^ −0.9 points for MDS‐UPDRS Part IV^35^ and −3.44 for PDSS‐2.^44^ *Scores are: 0: Normal (no OFF time); 1: Slight (≤ 25% of the waking day); 2: Mild (26–50% of the waking day); 3: Moderate 51–75% of the waking day; 4: Severe (>75% of the waking day).

### Patient‐ and Clinician‐Reported Outcomes

Quality of life PDQ‐8 summary index scores were improved from the initial values at both V2 and V3 (Table 2), and the improvement exceeded the minimal clinically important difference threshold (−5.49).^37^

Total scores for patient‐reported satisfaction with the LECIG infusion pump were high at both V2 and V3 (Table 3). Patients showed high satisfaction scores for all individual attributes of the pump (weight, size, noise, handling and overall satisfaction) at both V2 (mean scores >8.0/10 in each case) and V3 (mean scores >7.7/10 in each case). In terms of pump size and pump weight, all of the three subgroups (whether switched from LCIG infusion, APO infusion or a non‐infusion therapy) showed satisfactory outcomes with the LECIG pump when assessed at V2.

**TABLE 3:** Patient satisfaction scores for different attributes of the Crono® LECIG pump observed at visits 2 and 3. Values are mean ± standard deviation. Satisfaction scores for each item are rated on a 10‐point scale from 1 = absolutely dissatisfied to 10 = absolutely satisfied, except for the total satisfaction score which is a summary of all five items (a possible total score of 50)

Parameter	Observed at visit 2 (3–6 months)	Observed at visit 3 (6–12 months)
Pump size		
Did not switch from another infusion therapy	8.03 ± 2.07 (*n* = 137)	7.68 ± 2.14 (*n* = 72)
Switched from LCIG infusion	9.08 ± 1.08 (*n* = 12)	9.00 ± 1.73 (*n* = 3)
Switched from subcutaneous apomorphine infusion	6.00 ± 2.00 (*n* = 7)	5.00 ± 1.41 (*n* = 2)
Pump weight		
Did not switch from another infusion therapy	7.98 ± 2.09 (*n* = 137)	7.94 ± 2.13 (*n* = 72)
Switched from LCIG infusion	9.17 ± 1.11 (*n* = 12)	10.00 ± 0 (*n* = 3)
Switched from subcutaneous apomorphine infusion	7.00 ± 1.53 (*n* = 7)	6.00 ± 2.83 (*n* = 2)
Pump noise		
Did not switch from another infusion therapy	9.50 ± 1.86 (*n* = 137)	9.60 ± 0.94 (*n* = 72)
Switched from LCIG infusion	9.50 ± 1.45 (*n* = 12)	10.00 ± 0 (*n* = 3)
Switched from subcutaneous apomorphine infusion	9.00 ± 1.91 (*n* = 7)	7.00 ± 2.83 (*n* = 2)
Handling of the pump		
Did not switch from another infusion therapy	8.26 ± 1.80 (*n* = 137)	8.04 ± 2.04 (*n* = 72)
Switched from LCIG infusion	7.67 ± 2.35 (*n* = 12)	7.00 ± 1.73 (*n* = 3)
Switched from subcutaneous apomorphine infusion	7.86 ± 1.57 (*n* = 7)	5.00 ± 1.41 (*n* = 2)
Overall satisfaction with pump		
Did not switch from another infusion therapy	8.17 ± 1.83 (*n* = 137)	8.10 ± 1.88 (*n* = 72)
Switched from LCIG infusion	8.00 ± 2.37 (*n* = 12)	8.33 ± 1.53 (*n* = 3)
Switched from subcutaneous apomorphine infusion	7.57 ± 2.30 (*n* = 7)	5.00 ± 2.83 (*n* = 2)
Total satisfaction score (summary of all five items; possible total score of 50)		
Did not switch from another infusion therapy	41.93 ± 6.81 (*n* = 137)	41.36 ± 6.75 (*n* = 72)
Switched from LCIG infusion	43.42 ± 5.84 (*n* = 12)	44.33 ± 4.73 (*n* = 3)
Switched from subcutaneous apomorphine infusion	37.43 ± 4.69 (*n* = 7)	28.00 ± 8.49 (*n* = 2)

Abbreviation: LCIG, levodopa–carbidopa intestinal gel.

PGI‐I scores were improved when tested against a mean score of 4.0 (no change) at both V2 (2.28 ± 1.00; *n* = 159) and V3 (2.26 ± 1.00; *n* = 76; *P* < 0.0001 in each case). The treating clinicians reported similar improvements based on the CGI‐I at both V2 (2.16 ± 1.13; *n* = 154) and V3 (2.04 ± 1.11; *n* = 71; *P* < 0.0001 in each case).

### Safety

Table 4 provides an overall summary of observed AEs and summarizes the treatment‐emergent AEs that occurred in three or more LECIG‐treated patients and SAEs that occurred in two or more patients. Overall, 83 patients (49.7%; *n* = 167) reported a treatment‐emergent AE that occurred before or at V3. The most common AEs were procedure or device‐related, as expected. The list of all AEs that occurred in the Safety Set are detailed in Table S2. Further narrative detail on the AEs reported below can be found in S1.

**TABLE 4:** Summary of treatment‐emergent adverse events that occurred in LECIG‐treated patients at or before Visit 3 (*n* = 167)

Adverse event (AE)	Total, *n* (%)
Overall summary of AEs	
Any AE	83 (49.7%)
Serious AEs	50 (29.9%)
AEs leading to drug withdrawal in this dataset (patients who completed at least one post‐baseline visit)	3 (1.8%)
Device‐related AEs	
Total	28 (16.8%)
Serious	19 (11.4%)
Procedure‐related AEs	
Total	26 (15.6%)
Serious	15 (9.0%)
Drug‐related AEs	
Total	23 (13.8%)
Serious	11 (6.6%)
All AEs (serious and non‐serious) that occurred in at least three patients by MedDRA PT	
Device dislocation	20 (12.0%)
Device occlusion	5 (3.0%)
Stoma site infection	5 (3.0%)
Stoma site inflammation	5 (3.0%)
Weight decreased	5 (3.0%)
Diarrhea	4 (2.4%)
Hip fracture	3 (1.8%)
Hallucination	3 (1.8%)
Hallucination, visual	3 (1.8%)
Peritonitis	3 (1.8%)
Serious AEs that occurred in at least two patients by MedDRA PT*	
Device dislocation	15 (9.0%)
Device occlusion	3 (1.8%)
Diarrhea	3 (1.8%)
Hip fracture	3 (1.8%)
Peritonitis	2 (1.2%)
Pneumoperitoneum	2 (1.2%)
Stoma site inflammation	2 (1.2%)
Urinary tract infection	2 (1.2%)
Visual hallucination	2 (1.2%)

Abbreviations: MedDRA, Medical Dictionary for Regulatory Activities; PT, preferred term.

* Serious adverse events are a subset of all adverse events.

There were three patients in whom AEs led to withdrawal of LECIG treatment. The first was reported earlier, when delusion led to withdrawal of LECIG and subsequently study discontinuation. Two other patients only temporarily withdrew from LECIG treatment, and thus continued study participation: one reported device dislocation, the other reported two separate AEs of blood loss/anemia and duodenal ulcer.

Symptomatic diarrhea unrelated to LECIG treatment occurred in four patients (2.4%), and was of mild severity in one patient, moderate in two and severe in one. All patients continued with LECIG treatment. Dyskinesia was reported in two patients (1%) with moderate severity. Vitamin B12 deficiency was reported in two patients. One patient was reported to have clinically apparent peripheral sensory neuropathy which was first noted approximately 3 months from treatment initiation with LECIG. No further differentiation—axonal vs. demyelinating—was available.

In terms of psychiatric adverse effects acute psychosis was reported by one patient (0.5%). In addition, one patient was reported with “delusion”, while further AE annotations suggested that this patient also had psychotic symptoms. Another patient was reported with “neuropsychiatric symptoms” without further specification that resolved after adding clozapine. Hallucination was reported by seven patients (4%) and was resolved in six patients, while the outcome was unknown in one.

## Discussion

The ongoing ELEGANCE study is the largest of its kind to evaluate treatment outcomes with LECIG in patients with advanced PD in a real‐world setting. This interim analysis supports the clinically relevant efficacy of LECIG on PD motor symptoms that was sustained up to 1 year of treatment, as well as its positive impact on sleep and quality‐of‐life measures. LECIG treatment was well tolerated, and no new safety signals were observed. Importantly, patient‐ and clinician‐reported outcomes showed favorable satisfaction with the pump device.

Two multinational registry studies have previously reported on real‐world treatment outcomes with LCIG: GLORIA as a 24‐month observational study collected long‐term clinical outcomes in a large cohort of 375 patients and evaluated effects on motor and non‐motor symptoms and their impact on quality of life over 24 months.^38^, ^39^ DUOGLOBE was a 3‐year prospective, observational, multinational study of the efficacy and safety of LCIG treatment in 195 patients.^40^ Both studies showed findings comparable to this study regarding motor outcome, including reduction in daily hours of OFF time. It also compares to APO infusion therapy that has shown favorable stabilization of motor fluctuations both in a randomized controlled trial and real‐world longitudinal studies.^7^, ^8^, ^9^

LECIG was well tolerated for up to 1 year of treatment with a low rate of drug‐related AEs. Overall, the proportion of patients who experienced a treatment emergent AE in the ELEGANCE patient population (49.7%) aligned with that observed in the GLORIA study of LCIG treatment at 12 months (47.2%).^38^, ^39^ Similar to GLORIA, most AEs in the ELEGANCE analysis were related to the surgical procedure, stoma, and percutaneous endoscopic gastro‐jejunal (PEG‐J) tube rather than to drug treatment.

Diarrhea is a recognized drug‐related side effect of entacapone and occurs in around 10% of patients when taken orally.^21^, ^27^, ^41^ Here, diarrhea occurred rarely (2.4% of patients), and in all patients it was unrelated to LECIG treatment. The absence of entacapone‐related diarrhea in this cohort may mainly reflect the fact that patients were selected for study inclusion according to the LECIG SmPC, when there was no former history of diarrhea with oral entacapone.

The rate of peritonitis (1.8%) was low and no patient withdrew from LECIG for that reason. Further, it compares to the rates observed in patients undergoing PEG feeding procedures^42^ and to those reported in patients who have had PEG‐J placement for LCIG treatment,^43^ therefore most likely representing a procedure‐related adverse event.

Sleep problems are a common non‐motor symptom of PD. When assessed using PDSS‐2 scores as an outcome measure, the threshold for detecting a minimal clinically important difference in individual domains is considered to be −3.44 points for improvement and + 2.07 points for worsening.^44^ In our study, all PDSS‐2 domains improved substantially from baseline, the best improvement being seen in “disturbed sleep” at −2.92 points, approaching the recognized threshold value. In addition, it compares to findings from other continuous therapies which have demonstrated similar benefit on sleep quality when administered over 24 h.^36^, ^45^

LECIG treatment was observed to have a positive impact on quality‐of‐life measures, assessed using PDQ‐8 summary index scores with reductions of −13.3 and −11.1 points at V2 and V3, respectively. Notably, these reductions were greater than reported estimates for determining a minimal clinically important improvement in PDQ‐8 summary index scores of –5.94.^37^, ^46^

Health inequality in PD is an ongoing concern and recently, it has been discussed that COMT polymorphisms may differ across ethnic groups and impact on COMT inhibition therapy.^12^ Another study specifically studied the pharmacokinetics of levodopa with and without entacapone and found that entacapone improved levodopa bioavailability regardless of the common rs4680 COMT genotype. However, levodopa clearance tended to be higher in those with higher COMT activity.^14^ While this suggests that the majority of patients would be amenable to COMT inhibition with entacapone, there may be gradual differences in individual response patterns. Since our study consisted mostly of Caucasian patients, we could not perform additional statistical sub‐analyses to further evaluate this.

Another COMT inhibitor, opicapone, has been evaluated when given orally in combination with LCIG infusion.^13^ The study observed that oral opicapone led to LCIG daily dose being reduced by around 25% compared with LCIG monotherapy, which was suggested to lead to overall cost savings. A study by Senek et al showed that the dose reduction when using entacapone continuous infusion with LECIG was 35% and without the need for additional oral medications.^14^

While every effort has been made to standardize study procedures, as a registry‐based, real‐world study, ELEGANCE has some inherent limitations, in particular possible center differences in clinical practice and data collection which may influence treatment outcomes. While we were able to record the vast majority of study visits and measurements prospectively, V1 measurements were collected retrospectively in seven patients but referring to the time period prior to start of treatment with LECIG.

Assessments of OFF time were performed by the clinician who requested from the patient the daily hours spent in OFF from the corresponding MDS‐UPDRS part IV item. Patients may record their OFF times more precisely when compared to their dyskinetic ON times.^47^ However, this finding was revealed from prospective validation work on the Hauser diary and is not the methodology used in our study where we asked patients to recall their OFF times with the corresponding MDS‐UPDRS IV item.

Similarly, dyskinetic ON times were expressed as hours per day spent with dyskinesia according to MDS‐UPDRS IV item 4.1. However, this did not allow for further differentiation of “ON time with troublesome dyskinesia” versus “‘ON‐time with non‐troublesome dyskinesia” limiting comparison with randomized trials of device‐aided therapies.^6^, ^8^

The longitudinal outcomes from a small number of patients (*n* = 22) who switched from LCIG or APO infusion therapy are interpreted with caution and purely exploratory intent and should not be understood as a direct comparison. In particular, it is likely that factors like tolerability, compliance, hardware issues, and personal preferences for the pump device accounted for treatment failure and change to LECIG. Importantly however, we are not claiming superiority of one treatment over the other, which could only be evaluated in a head‐to‐head randomized controlled study. Descriptively, the lower satisfaction scores for the Crono® LECIG pump device for patients who switched from APO infusion to LECIG (*n* = 8) compared to switches from other therapies, might reflect the fact that the APO infusion pump, although of similar dimensions, is lighter than the Crono® LECIG pump (127 g versus 227 g, respectively).

In conclusion, the interim analysis of the ELEGANCE real‐world registry points to favorable clinical outcomes with relevant reduction in OFF‐time, increase in quality of life, and a low rate of bothersome dyskinesia and neuropsychiatric complications. AEs were mostly related to the PEG‐J system. As data accumulate on the real‐world use of LECIG and also other evolving medication therapies like subcutaneous foslevodopa/foscarbidopa therapy,^48^ it is likely that LECIG will find its position in patients in whom dopaminergic motor fluctuations impact quality of life and activities of daily living. Although intestinal therapies have sometimes been considered for the more advanced PD patients, in real‐world practice there are good reasons not to delay their use for too long until a late disease stage has been reached. Clinical real‐world practice suggests that if applied too late they may fail to reproduce the excellent findings of the randomized controlled trials and registry data reported for LCIG^49^ and as suggested for LECIG from the present ELEGANCE interim findings.

## Author Roles

(1) Research project: A. Conception, B. Organization, C. Execution. (2) Statistical Analysis: A. Design, B. Execution, C. Review and Critique. (3) Manuscript Preparation: A. Writing of the first draft, B. Review and Critique.

D.W.: 1A, 1C, 2C, 3A, 3B.

W.H.J.: 1C, 3B.

J.A.S.: 1C, 3B.

Z.P.: 1C, 3B.

I.M.: 1C, 3B.

V.T.: 1C, 3B.

N.K.: 1C, 3B.

H.S.: 2A, 2B, 2C, 3B.

B.A.: 1B, 1C, 2C, 3B.

N.S.: 1B, 1C, 2C, 3B.

T.V.L.: 1C, 3B.

## Disclosures

**Ethical Compliance Statement:** The study was conducted in accordance with principles for human experimentation as defined in the Declaration of Helsinki and International Conference on Harmonization Good Clinical Practice guidelines, and approved by all the relevant institutional review boards in each country. Informed consent was obtained from each study participant after they were told of the potential risks and benefits as well as the investigational nature of the study. We confirm that all authors have read and complied with the Journal's Ethical Publication Guidelines and have read the Journal's position on issues involved in ethical publication and affirm that this work is consistent with those guidelines.

**Financial disclosures related to this article:** The ELEGANCE study is sponsored by Britannia Pharmaceuticals Ltd. Funding for editorial assistance during manuscript development was provided by Britannia Pharmaceuticals Ltd. DW, WHJ, JAS, ZP, NK and TvL are paid by Britannia Pharmaceuticals Ltd. as members of the ELEGANCE study steering committee. HS is a statistical consultant for Britannia Pharmaceuticals Ltd. BA and NS are employees of Britannia Pharmaceuticals Ltd. The remaining authors have no conflicts of interest relevant to this work to declare.

**Financial disclosures for the previous 12 months:** DW has received honoraria as a speaker and consultant, and research grants from AbbVie, Abbott, Bial, Boston Scientific, Medtronic, Kyowa Kirin, and STADA. WHJ has received consultancy fees for acting as an advisor and speaker for AbbVie, Britannia Pharmaceuticals Ltd/STADA, UCB and Zambon. JAS has received consultancy and speaking honoraria from AbbVie, Boehringer‐Ingelheim, GSK, Lundbeck, Novartis, Pfizer, STADA, Teva and UCB. ZP has received speaker and consultant honoraria from Biogen, AbbVie, Britannia, STADA. IM has received honoraria for lectures from Teva Pharmaceutics, AbbVie, Britannia Pharmaceuticals Ltd, Pfizer, Hoffmann‐La Roche, Amgen, Swix, Ewopharma, Novartis, GL Pharma, Merck, and Medison. VT has received speaker and consultant honoraria from STADA and AbbVie. NK has received consultancy fees from Hungarian subsidiaries of STADA/Britannia Pharmaceuticals Ltd, AbbVie, Medtronic, Boston Scientific, Abbott, UCB, Krka, Richter and Medis; Advisory Board fees from AbbVie, Abbott, Boston Scientific, Britannia Pharmaceuticals Ltd. HS has acted as a statistical consultant for Norgine and US World Meds. BA and NS were paid employees of Britannia Pharmaceuticals Ltd. TvL has received study support from UMCG, Weston Brain Institute, Dutch Brain Foundation, MJFF and Menzis; lecture fees from AbbVie, Ever Pharma, Genilec and Britannia Pharmaceuticals Ltd.; Advisory Board fees from Britannia Pharmaceuticals Ltd, Centrafarm, BRC, Neuroderm, LTI, AbbVie and Ever Pharma.

## Supporting information


**Data S1.** Additional detail on some reported adverse events.

**Table S1.** Change from baseline in daily hours of OFF time following LECIG treatment at Visit 2 (3–6 months of treatment) in patients who switched from either apomorphine infusion or LCIG infusion.

**Table S2.** All treatment‐emergent adverse events reported for LECIG‐treated patients at or before Visit 3.

## Data Availability

The data that support the findings of this study are available from the corresponding author upon reasonable request.
