# Supplementary material for: Levodopa–Entacapone–Carbidopa Intrajejunal Infusion in Advanced Parkinson's Disease – Interim Analysis of the ELEGANCE Study
Source: Mov Disord Clin Pract. 2025 Mar 25;12(8):1075–85. doi: 10.1002/mdc3.70046 (PMC12371452; doi:10.1002/mdc3.70046)
Supplement: Supplementary file 1 — Data S1. Additional detail on some reported adverse events. [file MDC3-12-1075-s002.docx]

**SUPPLEMENTARY INFORMATION 1: Additional detail on some reported adverse events**

**Symptomatic diarrhea occurred in four patients (2.4%), and was of mild severity in one patient, moderate in two and severe in one.**

Additional information: In two patients an infectious reason unrelated to LECIG treatment was assumed and diarrhea resolved completely in both cases after antibiotic treatment; LECIG was then continued/re-initiated thereafter without re-emergence of diarrhea. One patient reported one diarrheic stool upon antibiotic treatment unrelated to LECIG treatment, which resolved spontaneously and did not re-occur under LECIG treatment. One patient reported diarrhea after feeding had been provided erroneously through the jejunal catheter at home. Diarrhea resolved after in-clinic management of the patient.

**Dyskinesia was reported in two patients (1%) with moderate severity.**

Additional information: One patient was admitted to in-hospital treatment with dyskinesia, which developed after the patient had administered two extra-doses of LECIG and in addition used oral levodopa-carbidopa-entacapone and 5 mg of selegiline. After in-clinic treatment this was resolved upon clinic discharge. The other reported dyskinesia increase occurred several weeks after hospital discharge, and this resolved after flow rate of LECIG was reduced. In addition, one patient was reported with an increase in ‘ON and OFF phenomenon’ and treated in clinic. The patient was reported to ‘be calmer’ upon reduction of the LECIG infusion dosages, which suggests dyskinesia albeit not explicitly reported. At the last available follow-up, the problem remained unresolved.

**Peripheral neuropathy**

One patient was reported to have clinically apparent peripheral sensory neuropathy which was first noted approximately 3 months from treatment initiation with LECIG. Vitamin B12 levels were reported in the normal range, homocysteine levels were not available, causality was judged ‘related’, and the outcome as ‘unresolved’. LECIG treatment was continued thereafter.

**Vitamin B12 deficiency was reported in two patients.**

Additional information: One patient had reduced vitamin B12 levels upon laboratory exam, and vitamin B12 supplementation was initiated without further follow-up laboratory examinations available. The other patient was reported with ‘functional vitamin B12 hypovitaminosis’ who showed vitamin B12 levels in the lower range (348 pg/ml, holotranscobalamin normal, methyl malonic acid elevated). No action was taken (it is unknown whether the subject had vitamin B12 supplementation previously) and no follow-up laboratory exams were available. Outcomes were indicated as unresolved in both patients.

**In terms of psychiatric adverse effects acute psychosis was reported by one patient (0.5%).**

Additional information: In the patient who experienced psychosis, this resolved after providing ‘concomitant medication’ with no further specifications available. In addition, one patient was reported with ‘delusion’, while further AE annotations suggested that this patient also had psychotic symptoms. Medication with LECIG was discontinued. No further follow-up was available. Another patient was reported with ‘neuropsychiatric symptoms’ without further specification that resolved after adding clozapine.

**Hallucination was reported by seven patients (4%).**

Additional information: In two patients the LECIG dose was changed, one unknown, others unchanged. Four patients received concomitant antipsychotic medication, i.e. quetiapine in three, and clozapine in one. In one patient, concomitant pramipexole treatment was discontinued.
