# Supplementary material for: Levodopa–Entacapone–Carbidopa Intrajejunal Infusion in Advanced Parkinson's Disease – Interim Analysis of the ELEGANCE Study
Source: Mov Disord Clin Pract. 2025 Mar 25;12(8):1075–85. doi: 10.1002/mdc3.70046 (PMC12371452; doi:10.1002/mdc3.70046)
Supplement: Supplementary file 2 — Table S1. Change from baseline in daily hours of OFF time following LECIG treatment at Visit 2 (3–6 months of treatment) in patients who switched from either apomorphine infusion or LCIG infusion. [file MDC3-12-1075-s003.docx]

**Supplementary Table 1:** Change from baseline in daily hours of OFF time following LECIG treatment at Visit 2 (3–6 months of treatment) in patients who switched from either apomorphine infusion or LCIG infusion.

| Parameter | Pre-LECIG treatment value | Change from pre-LECIG treatment at visit 2  (3–6 months) |
| --- | --- | --- |
| Daily hours of OFF time (mean ± SD score for MDS-UPDRS part IV, question 4.3) | | |
| Switched from apomorphine infusion (n=8) | 6.25 ± 4.73 (n=6) | -5.60 ± 5.59 (n=5) |
| Switched from LCIG infusion (n=14) | 3.75 ± 2.55 (n=10) | -1.38 ± 4.54 (n=8) |

LCIG, levodopa–carbidopa intestinal gel; LECIG, levodopa–carbidopa–entacapone intestinal gel; MDS-UPDRS, Movement Disorder Society Unified Parkinson's Disease Rating Scale; SD, standard deviation.
