# Supplementary material for: Levodopa–Entacapone–Carbidopa Intrajejunal Infusion in Advanced Parkinson's Disease – Interim Analysis of the ELEGANCE Study
Source: Mov Disord Clin Pract. 2025 Mar 25;12(8):1075–85. doi: 10.1002/mdc3.70046 (PMC12371452; doi:10.1002/mdc3.70046)
Supplement: Supplementary file 3 — Table S2. All treatment‐emergent adverse events reported for LECIG‐treated patients at or before Visit 3. [file MDC3-12-1075-s001.docx]

**Supplementary Table 2.** All treatment-emergent adverse events reported for LECIG-treated patients at or before Visit 3 (n=167).

| Primary system organ class | Total |
| --- | --- |
| Preferred term | (N=167) |
|  |  |
| Patients with events (%) - events | 83 (49.7%) - 179 |
|  |  |
| Product issues | 29 (17.4%) - 34 |
| Device dislocation | 20 (12.0%) - 25 |
| Device occlusion | 5 (3.0%) - 5 |
| Device malfunction | 2 (1.2%) - 2 |
| Device connection issue | 1 (0.6%) - 1 |
| Device defective | 1 (0.6%) - 1 |
|  |  |
| Infections and infestations | 23 (13.8%) - 27 |
| Stoma site infection | 5 (3.0%) - 6 |
| Peritonitis | 3 (1.8%) - 3 |
| Cellulitis | 2 (1.2%) - 2 |
| COVID-19 | 2 (1.2%) - 2 |
| Injection site infection | 2 (1.2%) - 2 |
| Pneumonia | 2 (1.2%) - 2 |
| Urinary tract infection | 2 (1.2%) - 2 |
| Helicobacter infection | 1 (0.6%) - 1 |
| Nasopharyngitis | 1 (0.6%) - 1 |
| Oral candidiasis | 1 (0.6%) - 1 |
| Orchitis | 1 (0.6%) - 1 |
| Pneumonia aspiration | 1 (0.6%) - 1 |
| Respiratory tract infection | 1 (0.6%) - 1 |
| Stoma site abscess | 1 (0.6%) - 2 |
|  |  |
| Gastrointestinal disorders | 18 (10.8%) - 21 |
| Diarrhoea | 4 (2.4%) - 4 |
| Abdominal pain, upper | 2 (1.2%) - 2 |
| Gastric ulcer | 2 (1.2%) - 2 |
| Gastritis | 2 (1.2%) - 2 |
| Pneumoperitoneum | 2 (1.2%) - 2 |
| Constipation | 1 (0.6%) - 1 |
| Duodenal ulcer | 1 (0.6%) - 1 |
| Dysphagia | 1 (0.6%) - 1 |
| Gastric disorder | 1 (0.6%) - 1 |
| Gastroesophageal reflux disease | 1 (0.6%) - 1 |
| Hemoperitoneum | 1 (0.6%) - 1 |
| Nausea | 1 (0.6%) - 1 |
| Pancreatitis | 1 (0.6%) - 1 |
| Salivary hypersecretion | 1 (0.6%) - 1 |
|  |  |
| Injury, poisoning and procedural complications | 17 (10.2%) - 21 |
| Stoma site inflammation | 5 (3.0%) - 5 |
| Hip fracture | 3 (1.8%) - 3 |
| Gastrostomy tube site complication | 2 (1.2%) - 2 |
| Device use issue | 1 (0.6%) - 1 |
| Fall | 1 (0.6%) - 1 |
| Femur fracture | 1 (0.6%) - 1 |
| Hand fracture | 1 (0.6%) - 1 |
| Procedural pain | 1 (0.6%) - 1 |
| Radius fracture | 1 (0.6%) - 1 |
| Skin laceration | 1 (0.6%) - 1 |
| Stoma complication | 1 (0.6%) - 1 |
| Stoma site erythema | 1 (0.6%) - 1 |
| Stoma site hypergranulation | 1 (0.6%) - 1 |
| Stoma site pain | 1 (0.6%) - 1 |
|  |  |
| Psychiatric disorders | 14 (8.4%) - 15 |
| Hallucination | 3 (1.8%) - 3 |
| Hallucination, visual | 3 (1.8%) - 3 |
| Sleep disorder | 2 (1.2%) - 2 |
| Acute psychosis | 1 (0.6%) - 1 |
| Anxiety disorder | 1 (0.6%) - 1 |
| Delusion | 1 (0.6%) - 1 |
| Depressed mood | 1 (0.6%) - 1 |
| Depression | 1 (0.6%) - 1 |
| Hallucination, auditory | 1 (0.6%) - 1 |
| Neuropsychiatric symptoms | 1 (0.6%) - 1 |
|  |  |
| Metabolism and nutrition disorders | 10 (6.0%) - 12 |
| Folate deficiency | 2 (1.2%) - 2 |
| Vitamin B12 deficiency | 2 (1.2%) - 2 |
| Decreased appetite | 1 (0.6%) - 1 |
| Dehydration | 1 (0.6%) - 1 |
| Diabetes mellitus | 1 (0.6%) - 1 |
| Hypercholesterolaemia | 1 (0.6%) - 1 |
| Hyperphagia | 1 (0.6%) - 1 |
| Hyperuricaemia | 1 (0.6%) - 1 |
| Hypervitaminosis B12 | 1 (0.6%) - 1 |
| Iron deficiency | 1 (0.6%) - 1 |
|  |  |
| Nervous system disorders | 9 (5.4%) - 10 |
| Bradykinesia | 2 (1.2%) - 2 |
| Dyskinesia | 2 (1.2%) - 2 |
| Dystonia | 1 (0.6%) - 1 |
| Freezing phenomenon | 1 (0.6%) - 1 |
| Hypercapnic coma | 1 0.6%) - 1 |
| Ischaemic stroke | 1 (0.6%) - 1 |
| ON and OFF phenomenon | 1 (0.6%) - 1 |
| Peripheral sensory neuropathy | 1 (0.6%) - 1 |
|  |  |
| Investigations | 9 (5.4%) - 9 |
| Weight decreased | 5 (3.0%) - 5 |
| Blood glucose increased | 1 (0.6%) - 1 |
| C-reactive protein increased | 1 (0.6%) - 1 |
| Electrocardiogram abnormal | 1 (0.6%) - 1 |
| Gamma-glutamyltransferase increased | 1 (0.6%) - 1 |
|  |  |
| General disorders and administration site conditions | 5 (3.0%) - 5 |
| Inflammation | 2 (1.2%) - 2 |
| Application site erythema | 1 (0.6%) - 1 |
| Drug ineffective | 1 (0.6%) - 1 |
| Granuloma | 1 (0.6%) - 1 |
|  |  |
| Skin and subcutaneous tissue disorders | 3 (1.8%) - 4 |
| Contact dermatitis | 1 (0.6%) - 1 |
| Hyperhidrosis | 1 (0.6%) - 1 |
| Rash | 1 (0.6%) - 2 |
|  |  |
| Respiratory, thoracic and mediastinal disorders | 3 (1.8%) - 3 |
| Pulmonary embolism | 2 (1.2%) - 2 |
| Chronic obstructive pulmonary disease | 1 (0.6%) - 1 |
|  |  |
| Blood and lymphatic system disorders | 2 (1.2%) - 2 |
| Blood loss anaemia | 1 (0.6%) - 1 |
| Leukocytosis | 1 (0.6%) - 1 |
|  |  |
| Cardiac disorders | 2 (1.2%) - 2 |
| Angina pectoris | 1 (0.6%) - 1 |
| Tricuspid valve incompetence | 1 (0.6%) - 1 |
|  |  |
| Musculoskeletal and connective tissue disorders | 2 (1.2%) - 2 |
| Intervertebral disc protrusion | 1 (0.6%) - 1 |
| Joint stiffness | 1 (0.6%) - 1 |
|  |  |
| Neoplasms benign, malignant and unspecified (including cysts and polyps) | 2 ( 1.2%) - 2 |
| Pancreatic carcinoma | 1 (0.6%) - 1 |
| Prostate cancer | 1 (0.6%) - 1 |
|  |  |
| Eye disorders | 1 (0.6%) - 1 |
| Visual acuity reduced | 1 (0.6%) - 1 |
|  |  |
| Hepatobiliary disorders | 1 (0.6%) - 1 |
| Jaundice | 1 (0.6%) - 1 |
|  |  |
| Renal and urinary disorders | 1 (0.6%) - 1 |
| Nocturia | 1 (0.6%) - 1 |
|  |  |
| Reproductive system and breast disorders | 1 (0.6%) - 1 |
| Vulvovaginal dryness | 1 (0.6%) - 1 |
|  |  |
| Vascular disorders | 1 (0.6%) - 1 |
| Orthostatic hypotension | 1 (0.6%) - 1 |
